# Unconventional Yeasts Isolated from Chilean Honey: A Probiotic and Phenotypic Characterization

**DOI:** 10.3390/foods13101582

**Published:** 2024-05-20

**Authors:** Adrian Rodríguez Machado, Camila Mella Caro, John J. Hurtado-Murillo, Cristian J. Gomes Lobo, Rommy N. Zúñiga, Wendy Franco

**Affiliations:** 1 Department of Chemical Engineering and Bioprocesses, Pontificia Universidad Católica de Chile, Ave. Vicuña Mackenna 4860, Santiago 6904411, Chile; arodriguez17@uc.cl (A.R.M.); jdhurtado@uc.cl (J.J.H.-M.); cjgomes@uc.cl (C.J.G.L.); 2 Department of Biotechnology, Universidad Tecnológica Metropolitana, Las Palmas 3360, Ñuñoa, Santiago 7800003, Chile; c.mellac@utem.cl (C.M.C.); rommy.zuniga@utem.cl (R.N.Z.); 3 Department of Health Sciences, Nutrition Career, Pontificia Universidad Católica de Chile, Ave. Vicuña Mackenna 4860, Santiago 6904411, Chile

**Keywords:** probiotic yeasts, *in vitro* digestion, fermentation, honey, antimicrobial activity

## Abstract

This study explores the potential probiotic properties of yeasts isolated from various Chilean honeys, focusing on Ulmo, Quillay, and Mountain honeys. Six yeast strains were identified, including *Zygosaccharomyces rouxii*, *Candida* sp., *Schizosaccharomyces pombe*, *Rhodosporidiobolus ruineniae*, *Clavispora lusitaniae*, and *Metschnikowia chrysoperlae*. Phenotypic characterization involved assessing their fermentative performance, ethanol and hops resistance, and cross-resistance. Ethanol concentration emerged as a limiting factor in their fermentative performance. The probiotic potential of these yeasts was evaluated based on resistance to high temperatures, low pH, auto-aggregation capacity, survival in simulated *in vitro* digestion (INFOGEST method), and antimicrobial activity against pathogens like *Escherichia coli*, *Staphylococcus aureus*, and *Salmonella enteritidis*. Three yeasts, *Zygosaccharomyces rouxii*, *Schizosaccharomyces pombe*, and *Metschnikowia chrysoperlae*, exhibited potential probiotic characteristics by maintaining cell concentrations exceeding 10^6^ CFU/mL after *in vitro* digestion. They demonstrated fermentative abilities and resistance to ethanol and hops, suggesting their potential as starter cultures in beer production. Despite revealing promising probiotic and technological aspects, further research is necessary to ascertain their viability in producing fermented foods. This study underscores the innovative potential of honey as a source for new probiotic microorganisms and highlights the need for comprehensive investigations into their practical applications in the food industry.

## 1. Introduction

Probiotics are live microorganisms that, when consumed in adequate amounts, confer health benefits to the host [1]. They have traditionally been used in the production of dairy products; however, due to factors such as lactose and milk protein intolerance, and the need to control cholesterol and saturated fatty acid levels, new matrices are required to develop probiotic foods [2].

Within the criteria for selecting probiotic strains, resistance to gastrointestinal (GI) transit is crucial when the strain’s primary goal is to balance the intestinal microbiome [3]. Other characteristics that define the probiotic nature of yeasts include resistance to low pH, resistance to bile salts, and the ability to survive and growth at body temperature (37 °C) [4]. Although most tested characteristics of probiotic yeasts are assumed to vary and depend on the strain type, it is very challenging to find a probiotic yeast strain that meets all desirable functional properties. Therefore, the criteria for selecting potential probiotic candidates depend on the purpose of the product or food to be developed.

Unlike probiotic bacteria, probiotic yeasts have been relatively understudied. *S. boulardii* is the only patented probiotic yeast whose functionality has been demonstrated in double-blind studies [5]. This yeast has been used in foods such as yogurt, kefir milk [6], fermented cereal-based beverages [7], and corn-based cereals, among other foods [8], to alleviate digestive issues such as microbial imbalance, nausea, indigestion, abdominal bloating, ulcers, abdominal pain, and loss of appetite [9]. With an innovative perspective, yeasts are being used to help restore good strains of microorganisms and intestinal balance. They are also being considered for developing functional foods, which are increasingly valuable in the food industry [10].

From a functional standpoint, yeasts contribute to the stimulation of the host’s defense system immune modulation. On the other hand, they contribute to flavor formation in fermented foods, in addition to producing antimicrobial molecules, such as organic acids and antimicrobials [11], including acetic acid, lactic acid, formic acid, and short-chain fatty acids [12]. Moreover, killer yeast starters enhance food safety by inhibiting the growth of pathogens during fermentation, preventing the adherence of pathogenic microorganisms to the intestinal mucosa [13], and suppressing the proliferation of spoilage microorganisms [14].

Yeasts are ubiquitous and can be found in various ecological niches and products of natural origin. For example, bee honey possesses symbiotic properties, making it an excellent alternative source of prebiotics and probiotics [15]. It has been used to isolate yeasts with probiotic characteristics, given that its oligosaccharide content promotes the establishment of microorganisms [16]. The yeast diversity encounter in honeys is often associated with differences in floral sources, climate, and bee-related factors from where the honey is produced [16,17]. According to the Chilean Agricultural Research Institute (INIA), sugars’ characteristics, properties, and composition depend mainly on environmental, geographical, and botanical indicators. Therefore, the characteristics of honey produced in Chile differ from those in other regions [17]. Additionally, Chilean bee honey stands out for its high ash, potassium, zinc, and sucrose content and low sodium content [17]. These characteristics could promote the presence of yeast strains with potential probiotic characteristics.

Given that the food industry is continuously looking for new yeast strains that, beyond alcoholic fermentation, are able to give functionally to food products, we decide to characterize the allochthonous yeasts present in different Chilean honeys, given that honey has been reported as a reservoir for probiotic yeasts. Therefore, the aim of this study was to isolate yeasts from honey of Chilean origin and subsequently determine their probiotic potential in an *in vitro* semi-dynamic model (INFOGEST). Initially, yeasts were isolated from Ulmo honey (from *Eucryphia cordifolia*), Quillay honey (from *Quillaja saponaria*), and Mountain honey. These isolates were phenotypically identified and characterized, followed by an assessment of their fermentative performance and potential probiotic characteristics.

## 2. Materials and Methods

### 2.1. Samples

Three types of Chilean bee honey were used: monofloral Ulmo honey (*Eucryphia cordifolia*), which is endemic to Los Rios region (coordinates 39°48′30′ S 73°14′30′ O), monofloral Quillay honey (*Quillaja saponaria*), endemic to the central zone of Chile (parallel 38° S), and multifloral Mountain honey from Los Lagos region (coordinates 41°28′18′ S 72°56′12′ O). The certified honeys were obtained from the Natural Products Laboratory of the Faculty of Agronomy at the Pontifical Catholic University of Chile.

#### Isolation and Identification of Yeasts

The honey samples were serially diluted with 1% peptone water (Condalab, Madrid, Spain). To induce yeast growth, 1 mL of each dilution was inoculated into 20 mL of Sabouraud dextrose broth (SBD, Condalab, Madrid, Spain) [18]. The medium was incubated at 25 °C for two days. After this period, aliquots were plated on yeast glucose chloramphenicol agar (YGC, Condalab, Madrid, Spain) and incubated at 25 °C for 3–5 days or until the formation of colonies was observed. Colonies with the same morphology were randomly selected and stored at −20 °C.

For the identification of isolates, the YeaStar Genomic DNA Kit™ (Tustin, CA, USA) was used, following the procedure established by the supplier. Gene amplification was carried out in the D1/D2 domain of the 26S rRNA by polymerase chain reaction (PCR), using the primers ‘L-1 (5’-GCATATCAATAAGCGGAGGAAAAG) and ‘L-4 (5’-GGTCCGTGTTTCAAGACGG), as described by Kurtzman and Robnett [19]. The amplified DNA was visualized through electrophoresis on 1.5% agarose gel in 1X TBE buffer (89 mM Tris-acetate, 89 mM boric acid, 2 mM of ethylenediaminetetraacetic acid (EDTA). Sequencing reactions for both forward and reverse strands were performed by MACROGEN (Seoul, Republic of Korea).

The partial sequences of 26S rRNA obtained were analyzed using the Basic Local Alignment Search Tool (BLAST 2.2.26) algorithm [20], accessible on GenBank [21], using the non-redundant nucleotide database [22]. Only alignments with an identity match greater than 95% were considered for identification purposes.

### 2.2. Phenotypic Characterization of Isolated Yeasts

The isolated yeast strains were phenotypically characterized in synthetic media composed of glucose, maltose, ethanol, and hops, as shown in Table 1. Fermentations were conducted using malt extract (OXOID, Crawley, UK) as the base medium (glucose 11.9 g/L, fructose 2.8 g/L, maltose 58.2 g/L, maltotriose 12.7 g/L, and sucrose 1.5 g/L).

Each yeast’s active cultures (1 × 10^6^ CFU/mL) were inoculated (2%) into the different defined media. Fermentations were carried out in Erlenmeyer flasks (100 mL), equipped with airlocks and sampling probes, at 27 °C and 100 rpm (Shaker JSR, JSSI-100C, Gongju-Si, Republic of Korea) for 72 h. Samples were taken every 8 h to measure the optical density at 600 nm (OD_600_) using a spectrophotometer (UV/VIS Spectrophotometer, UV-M51, Belphotonics, Monza, Italy), and metabolites, including lactic acid, acetic acid, ethanol, sucrose, fructose, glucose, and glycerol, were determined by high-pressure liquid chromatography (HPLC).

#### Analysis of Metabolites and Substrates by High Pressure Liquid Chromatography (HPLC)

The concentrations of metabolites and substrates were measured by HPLC using a 30 cm HPX-87H column (Bio-Rad Laboratories, Hercules, CA, USA) for component separation. The column temperature was maintained at 37 °C, and the elution of components was performed with 0.03 N sulfuric acid at a flow rate of 0.6 mL/min. A Thermo Separations UV6000 diode array detector (Spectra System Thermo Scientific, Waltham, MA, USA) and a Waters model 410 refractive index detector (Waters Corp., Millipore Corp., Billerica, MA, USA), connected in series with the diode array detector, were used to measure glucose, fructose, ethanol, and other metabolites. External standardization of the detectors was performed using four concentrations of standard compounds [23].

### 2.3. Determination of Probiotic Capacity

The probiotic nature of yeasts is defined by resistance to low pH, survival and growth capacity at high temperatures (37 °C), tolerance to gastric acidity, resistance to bile salts, auto-aggregation capability, and resistance to the gastrointestinal tract. The latter is one of the most commonly used criteria for selecting probiotic strains to balance the intestinal microbiome.

#### Survival at Low pH and Growth at 37 °C

Six selected yeast strains underwent tests for tolerance at pH 3.5 and a temperature of 37 °C to select strains that exhibited resistance for further studies. Active cultures (1 × 10^6^ CFU/mL) were inoculated (2%) in malt extract broth (OXOID, Crawley, United Kingdom). The pH was adjusted to 3.5 only for this treatment using an acidic solution (1 M HCl, Merck, Germany) [24], and fermentation was conducted at 27 °C and 100 rpm (JRC) for 72 h. For temperature resistance, a malt extract (OXOID) medium was used and incubated at 37 °C and 100 rpm for 72 h. Samples were taken every 8 h to measure OD_600_ and obtain a growth curve for each microorganism and treatment conducted.

### 2.4. Aggregation Capacity

The yeast isolates (1 × 10^6^ CFU/mL) were cultured in 10 mL of SBD broth (Condalab) at 37 °C for 24 h. After cultivation, the cells were centrifuged, washed, and resuspended in phosphate-buffered saline (PBS) solution (50 mmol L-1 K_2_ HPO_4_/KH_2_ PO_4_, pH 6.5). The resulting cell suspension was vortexed in 3 mL of the same solution, and then 1 mL of the suspension was transferred to a plastic cuvette. Cell auto-aggregation was determined by measuring the optical density OD_600_ nm [25] using a spectrophotometer (UV-M51) after 12 and 24 h of incubation at 37 °C, following the methodology described by Gil-Rodríguez et al. [25]. To determine the percentage of auto-aggregation, the following expression was used:

(1) $$\%\;\text{Autoagregación}=\left[1-\left(A_{t}/A_{0}\right)\right]*100\%$$

where *A_t_* is the absorbance at 12 h and 24 h, and *A*_0_ is the absorbance at time zero.

### 2.5. In Vitro Digestion

The *in vitro* digestion process was divided into oral, gastric, and intestinal stages. Simulated oral, gastric, and intestinal fluids, along with pH and temperature conditions, were employed, following the protocol defined by INFOGEST [26], adapted to a semi-dynamic system [27].

The sample’s transit through the oral cavity was simulated during the initial phase. Subsequently, the second phase involved *in vitro* gastric digestion, using a double-jacketed beaker containing a gastric solution subjected to constant agitation. This solution simulated conditions present in the human stomach. Finally, in the third phase, the *in vitro* intestinal digestion was performed by conditioning the chyme obtained from the gastric phase to allow its passage through the small intestine.

#### 2.5.1. Preparation of Simulated Digestive Fluids

For the *in vitro* digestion assays, simulated oral fluids [17], simulated gastric fluids (SGF), and simulated intestinal fluids (SIF) were prepared. Table 2 details the stock solutions’ components and composition for preparing simulated fluids, and Table 3 shows the quantity and proportion of stock solutions and components in the simulated fluids. Before starting the SGF preparation, the stock solution’s pH was adjusted to pH 7.0 by adding 1N HCl. These stock solutions were prepared following the INFOGEST standard protocol for *in vitro* digestion [27].

#### 2.5.2. Oral Phase of In Vitro Digestion

Before the initiation of gastric digestion, 1.5 mL of oral stock solution (SSO) was added to the yeast cultures, with the volume of SSO being in relation to the solids content of the sample, based on the INFOGEST methodology. The mixture was maintained for 2 min at a temperature of 37 °C, simulating the passage through the oral cavity. In this instance, the SSO lacked α-amylase, since the experimental samples did not contain starch. The volume of OSS used was calculated in proportion to the amount of proteins present in the sample [27].

##### Curves of Change in Gastric pH

Before gastric digestion, gastric pH change curves were generated using the following procedure: 50 g of the sample at 37 °C were mixed with 45 mL of simulated fluid gastric (SFG). The pH of this mixture was monitored by an automatic titration system (Metrohm, 902 Titrando, Herisau, Switzerland) with the software (Tiamo^TM^ 2.4) adjusted to the necessary parameters for obtaining the expected pH curve. The study was based on adding defined volumes of a 1.5 N HCl solution at different time intervals.

The method involved continuously adjusting the pH of the sample undergoing gastric digestion throughout the entire test period (90 min). This adjustment aimed to incorporate an acidic solution and achieve a pH change curve similar to the reference curve obtained from the literature for *in vivo* digestions [28,29]. The procedure included triplicates of the gastric pH change curves.

#### 2.5.3. *In Vitro* Gastric Digestion

A specific basal secretion occurs in the human stomach, even in a fasting state [27]. For this reason, a basal gastric solution was prepared to simulate this condition, allowing the pH to decrease as soon as the sample was added to the simulated stomach. The basal gastric solution was designed so that the pH of the gastric content was higher than that of gastric secretion. This solution comprised 10% of the total volume of gastric secretion used in the *in vitro* digestion.

The experimental sample (from the oral phase) was added to a double-jacketed beaker with constant stirring at 60 rpm to initiate gastric digestion. The space between the glass walls of the beaker was filled with water at 37 °C, which was pumped using a peristaltic pump (Gilson, Miniplus Evolution, Middleton, WI, USA). Subsequently, SGF was added to the solution for 2 min, and then 5 mL of basal gastric solution was adjusted to pH 2.0 with 1N HCl. Once the time elapsed, gastric digestion was initiated.

For 90 min, 1.5 N HCl and 45 mL of SGF were added to the sample at different volumetric flows using syringe pumps (New Era Pump Systems Inc, NE-1000, Tampa, FL, USA). The pH was monitored with an automatic titrator (Metrohm, 902 Titrando, Herisau, Switzerland) controlled by the equipment software (Tiamo^TM^, Herisau, Switzerland). The pH was adjusted to obtain a pH curve similar to that *in vivo* found by Hoebler et al. [28] and Krul et al. [29]. The flows and volumes of 1.5 N HCl to be added to the gastric phase were determined from the data obtained in the *in vitro* pH curve assays.

During the simulated *in vitro* digestion, gastric emptying occurred, involving the passage of chyme from the stomach to the intestine through the pylorus, which acts as a filter, allowing the passage of liquids and particles smaller than 3 mm [27]. To replicate this physiological process, 20 mL of gastric chyme was collected at 18 min intervals during gastric digestion. The collected chyme was adjusted to pH 7.0 and used in the next stage of the simulation, corresponding to intestinal digestion. A peristaltic pump (Mini-Pump Flow Variable, Cole Palmer, Jinshan District, Shanghai, China), connected to an outlet at the bottom of the double-jacketed beaker, was used to recover the chyme. Once the gastric phase was completed, the chyme obtained was used in the subsequent simulation stage, corresponding to the intestinal phase.

#### 2.5.4. *In Vitro* Intestinal Digestion

The *in vitro* intestinal digestion was carried out using the gastric content obtained from each sample at different time intervals. For this stage, a simulated intestinal fluid solution (SIF) was prepared, as mentioned earlier, and its pH was adjusted to 7.0. The pH of the chyme was raised to 7.0 using 1N NaOH to simulate the conditions of the *in vivo* small intestine and to stop pepsin activity. Then, both liquids were mixed in a 1:1 ratio in 50 mL Falcon tubes and kept under continuous agitation at 80 rpm for 2 h at 37 °C, using a temperature-controlled shaker bath (Water Bath Shaker, SWBR17, Cornelius, NC, USA). After completing intestinal digestion, Pefabloc^®^ SC (4-(2-aminoethyl) benzenesulfonyl fluoride hydrochloride) (Sigma Aldrich, Schnelldorf, Germany) was added as an enzymatic inhibitor to stop the activity of pancreatin in the sample. Samples were taken and serial dilutions were made for cell counting to determine if the yeasts could survive the *in vitro* digestion.

### 2.6. Antimicrobial Activity Assays

Antimicrobial activity assays were conducted following the methodology proposed by Chelliah et al. [30] and Palande et al. [31]. Briefly, antimicrobial assays were performed against enteropathogens such as *Escherichia coli* (ATCC 35218TM), *Staphylococcus aureus* (ATCC BAA–1026TM), and *Salmonella enteritidis* (ATCC 49223TM). Pathogens were cultured on Mueller–Hinton agar plates (OXOID CM0337, Basingstoke, UK) and incubated at 37 °C for 10 min. White discs (Antimicrobial Susceptibility, OXOID, Basingstoke, UK) were used, and the assays were conducted by flooding the discs with the supernatant and the precipitate from the yeast culture. Tetracycline and ampicillin-containing discs were used as positive controls and white discs were used as negative controls.

The yeast isolates were cultured in SBD broth at 27 °C for 24 h. The supernatant was collected after centrifugation (Centrifuge Z 326K, Hermle, Wehingen, Germany) at 10,000 rpm for 20 min at room temperature, and 20 μL were added to the white discs. The precipitate was mixed with peptone water (1%), and 20 μL were added to the white discs. Prepared disks were placed on top of the agar plates containing the active bacterial cultures. The growth inhibition zones were measured after incubating the plates at 37 °C for 24 h. Antimicrobial activity was recorded as clear inhibition zones free of bacterial growth around the discs. The appearance of inhibition halos with a diameter greater than 5 mm (around each disc) was considered positive antagonistic activity.

### 2.7. Statistical Analysis and Plotting

All the experiments were carried out in triplicate in two experiment runs. The data were expressed as mean ± standard deviation. A one-way analysis of variance (ANOVA) was performed to determine significant differences. The post hoc *t*-test with Bonferroni correction was applied for multiple comparisons. Statistically significant differences were considered at *p* values of <0.05.

## 3. Results and Discussion

### 3.1. Identification of Yeasts Isolated from Honey Samples

Ten yeast isolates were obtained, resulting in six identified yeast strains belonging to different species. The isolated yeast strains were as follows: from Ulmo honey, *Zygosaccharomyces rouxii*, *Candida* sp., and *Schizosaccharomyces pombe*; from Mountain honey, *Rhodosporidiobolus ruineniae* and *Clavispora lusitaniae*; and from Quillay honey, *Metschnikowia chrysoperlae* (Table 4). These yeasts were morphologically characterized, and their cellular structures were observed under the microscope.

Few studies have reported yeast isolated from bee honey. Among the reported strains, those belonging to the genus *Saccharomyces* are the most prevalent. However, strains other than *Saccharomyces* have also been reported, including *Rhodotorula*, *Debaryomyces*, *Hansenula*, *Lipomyces*, *Oosporidium*, *Pichia*, *Torulopsis*, *Trichosporon*, *Nematospora*, *Schizosaccharomyces*, *Schwanniomyces*, *Torul*, and *Zygosaccharomyces* [32,33,34]. Other studies have reported the isolation of yeasts from bees themselves, and the reported species include *Saccharomyces cerevisiae*, *Meyerozyma guilliermondii*, and *Saccharomyces* var. *boulardii* [14].

In the research conducted by Sinacori et al. [18], the composition of cultivable microbial populations in 38 honey samples, both from nectar and honeydew, from various botanical and geographical sources, was analyzed. The results of this study indicate the identification of five yeast species, including *Aureobasidium pullulans*, *Cryptococcus uzbekistanensis*, *Debaryomyces hansenii*, *Zygosaccharomyces rouxii*, and *Zygosaccharomyces mellis*, which was the most prevalent species isolated. Although a greater microbial diversity was observed in multifloral honey, no significant correlation was found between microbial species and botanical or geographical origin.

In our study, we isolated yeasts not reported previously, and this diversity may be related to the specific characteristics of the honey used, associated with the regions’ geographical and climate characteristics.

### 3.2. Phenotypic Characterization of Isolated Yeasts

#### 3.2.1. Carbohydrate Utilization

**Figure 1:**
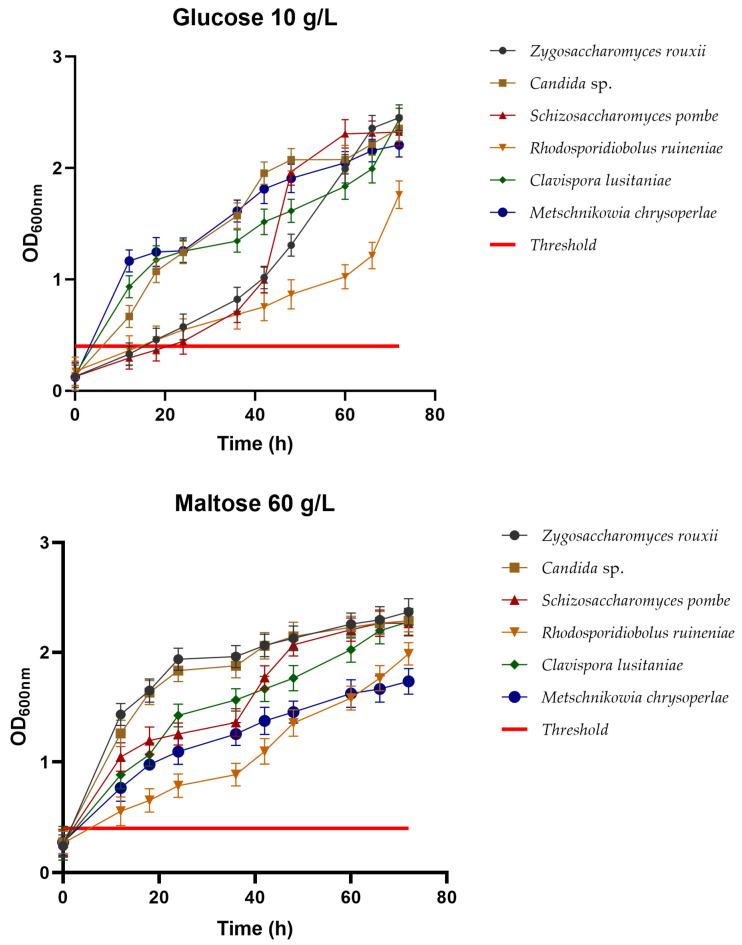
Cell growth (OD_600_) after 72 h of incubation at 25 °C of the six yeast strains isolated in synthetic media with glucose (10 g/L) and maltose (60 g/L). The growth threshold was established at 0.4; values above this point were considered to denote yeast growth, while values below the point were considered to represent the inability of the yeast to grow in the media.

Using carbohydrates, such as glucose and maltose, is essential when characterizing potential starter cultures for the food industry, for example, to produce alcoholic beverages like beer. The results of the yeast isolates’ metabolism are shown in Figure 1 and Table 5 and Table 6, representing the behavior of the yeasts *Z. rouxii*, *Candida* sp., *S. pombe*, *R. ruineniae*, *C. lusitaniae*, and *M. chrysoperlae* over a 72 h incubation period at a temperature of 27 °C and 150 rpm agitation. The growth threshold was defined at an optical density read at 600 nm (OD_600_) of 0.4 in the diagrams [35]; values higher than this indicate significant growth, while values below indicate that the yeasts have not experienced significant growth.

All six yeast strains can efficiently utilize both glucose and maltose sugars, as evidenced by the increase in OD_600_. In the case of growth in the defined glucose medium, the yeasts *Z. rouxii* with OD_600_ 2.45 and *C. lusitaniae* with OD_600_ 2.43 exhibit the highest cellular growth, compared to *R. ruineniae* with OD_600_ 1.76, which shows the lowest growth.

In the case of the defined maltose medium, the yeasts *Z. rouxii*, *Candida* sp., *S. pombe*, and *C. lusitaniae* exhibit very similar growth, reaching the highest values with OD_600_ 2.36, OD_600_ 2.28, OD_600_ 2.26, and OD_600_ 2.27, respectively. A different scenario was observed for *M. chrysoperlae*, which showed the lowest growth, with OD_600_ 1.73. The ability of the isolated yeast to grow in sugar-rich environments might be related to their osmotolerant ability; having been isolated from honey, these yeasts have an adaptive response to extracellular osmotic pressure, which allows them to pump ions from the cell exterior to the interior, and synthesize and concentrate various solutes (such as sugars, polyalcohols, amino acids, and glycerol) which allow them to maintain the cell integrity [36,37].

As part of the fermentative metabolism, yeasts are able to produce certain metabolites, and Table 5 shows the concentrations of glucose, ethanol, glycerol, lactic acid, and acetic acid at the end of the fermentation in the glucose-defined medium. *S. pombe* produced the highest ethanol concentration (2.26 ± 0.04% *v*/*v*), lactic acid concentration (0.23 ± 0.01 g/L), acetic acid concentration (0.59 ± 0.20), and was the second-highest producer of glycerol (1.56 ± 0.06 g/L). It consumed the highest amount of sugars compared to the other yeasts, with values of sucrose reaching 0.53 ± 0.03 g/L at the end of fermentation and consumed all of the fructose and glucose.

In the case of the medium supplemented with maltose (60 g/L), Table 6 shows the same behavior for the yeast *S. pombe*. The highest concentrations of lactic acid (0.27 ± 0.01 g/L) and acetic acid (0.74 ± 0.3 g/L) were obtained, but a higher concentration of ethanol (1.62 ± 0.06% *v*/*v*) was produced. Regarding sugars, the yeast consumed all glucose at the end of fermentation.

Little information is available associated with the fermentative metabolism of yeast isolated from honey. Matraxia et al. [35] reported the percentage of sugar consumption in various fermentation treatments using yeast isolated from fermented honey. Regarding glucose, the yeasts in that study demonstrated the ability to consume 99.40 ± 0.02%, while for fructose, they consumed 97.30 ± 0.05%. In the case of sucrose, the consumption range in that study varied from 37.90 ± 0.39% to 100 ± 0%. These results are consistent with ours, as the honey-isolated yeasts also consumed virtually all the glucose and, to a lesser extent, sucrose during the phenotypic characterization tests. Regarding the produced metabolites, the authors of the previously mentioned study indicate that the concentration of ethanol produced varied between 0.52 and 5.16% (*v*/*v*), while acetic acid ranged between 0.03 and 0.26 g/L. As for glycerol, the values fluctuated between 1.28 and 3.80 g/L. In our research, the ethanol ratio falls between 0.07 ± 0.008% and 2.26 ± 0.04%, with the maximum concentration being lower than reported by these authors. Regarding acetic acid, the yeasts studied in this work produced slightly higher concentrations than those reported in the previously mentioned work. In contrast, the concentrations of glycerol generated were lower than those mentioned by the previously cited authors. These differences might be attributed to the different species evaluated in our study; as they are non-*Saccharomyces* yeasts, they show a different metabolism.

In another study, Prestianni et al. [38] studied *Saccharomyces cerevisiae* and *Hanseniaspora uvarum* isolated from honey by-products to evaluate the influence of taste and olfactory attributes in mead. Regarding sugar consumption, the authors reported that, in various fermentation treatments, a residual glucose percentage ranging from 0.72 to 31.86 g/L was obtained, while for fructose, it was from 1.05 to 40.76 g/L. Compared to our results, the residual sugar concentration reported by these authors is higher than what we observed in our research, where these sugars were consumed almost entirely. Regarding the produced metabolites, the authors of the other work reported concentrations of acetic acid ranging from 0.29 ± 0.02 g/L to 0.71 ± 0.06 g/L, ethanol from 5.31 ± 0.81% to 12.37 ± 1.07%, and glycerol from 4.31 ± 0.14 g/L to 7.25 ± 0.66 g/L. When comparing these results with ours, we observe that, in the case of acetic acid, our concentrations range from 0.17 ± 0.03 g/L to 0.74 ± 0.30, obtaining similar results to those reported by the authors of the other study. The ethanol percentage obtained in our research is lower than that reported by these authors, possibly because they carried out sequential fermentation treatments. Regarding glycerol, we also obtained concentrations, in most cases, below those reported by these authors, possibly due to the use of different yeast strains with different metabolism.

#### 3.2.2. Ethanol Tolerance

**Figure 2:**
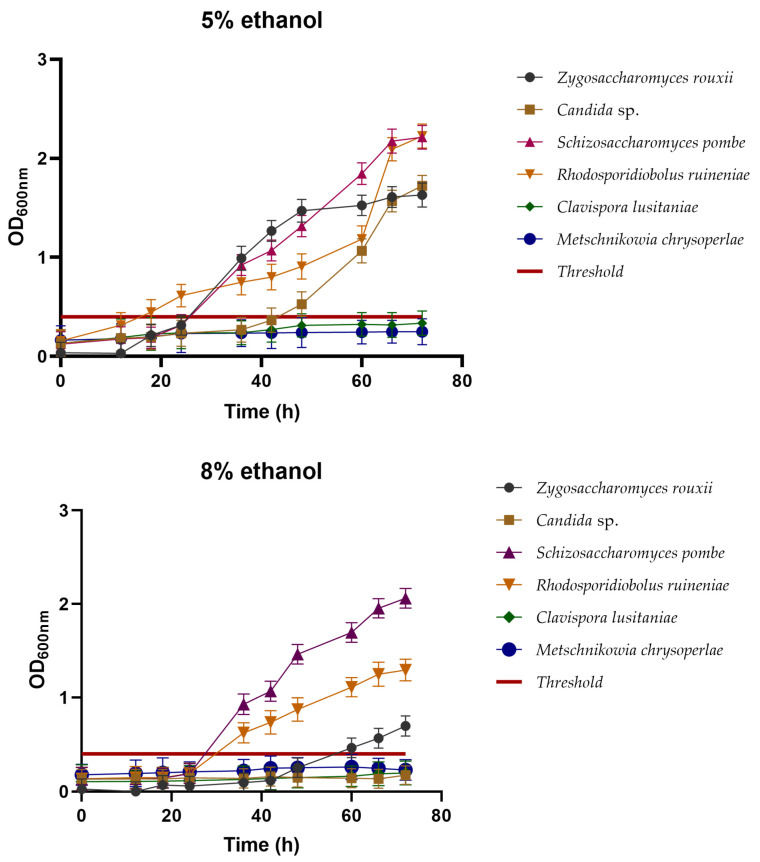
Tolerance assay in medium with ethanol at 5% (*v*/*v*) and 8% (*v*/*v*) after at 72 h of cell growth determined at 600 nm. Growth threshold established at 0.4. Values above this point were considered to denote yeast growth, while values below this point were considered to represent the inability of the yeast to grow in the media.

Ethanol tolerance was studied at two concentrations, 5% (*v*/*v*) and 8% (*v*/*v*), for the six yeast strains. Figure 2 shows the behavior of these yeasts under both treatments. The minimum growth threshold was set at an OD_600_ of 0.4.

In the case of the fermentation carried out at 5% (*v*/*v*), the yeasts *Z. rouxii*, *Candida* sp., *S. pombe*, and *R. ruineniae* exhibited good growth, easily surpassing the established growth threshold, with OD_600_ values of 1.52, 1.06, 1.84, and 1.18, respectively. However, the yeasts *Candida* sp. and *M. chrysoperlae* remained below the growth threshold, indicating that they are not tolerant to ethanol concentrations commonly reached in the fermentation process for beer making. Nevertheless, these yeasts could be considered for non-alcoholic fermentation or obtaining beverages with an ethanol concentration lower than 5% (*v*/*v*).

During fermentation at 8% (*v*/*v*), a lag phase of more than 20 h was observed for all the yeast isolates; after this time, increases in optical density were observed to finally reach values above DO_600_ of 2, which suggest that the yeast needed to become used to the ethanol-rich medium before increasing in cell concentration. However, only the yeasts *Z. rouxii*, *S. pombe*, and *R. ruineniae* exhibited optimal growth above the threshold, reaching OD_600_ values of 0.56, 1.69, and 0.90, respectively. Meanwhile, *Candida* sp., *C. lusitaniae*, and *M. chrysoperlae* were inhibited at this ethanol concentration.

In general, our results showed that the yeast *S. pombe* exhibited the best performance compared to others in both treatments regarding its fermentative metabolism and ethanol tolerance. This suggests that it is an interesting candidate for the alcoholic beverage industry. The yeast species has been previously reported as a starter culture for the production of wine. Interestingly, the utilization of the yeast allowed a deacidification of the wines without the use of lactic acid bacteria, given the yeast’s capacity to utilize malic acid along with the alcoholic fermentation [39,40,41]. García et al. [42] analyzed the ethanol resistance of the yeast *S. pombe*, isolated from the spontaneous fermentation of Malvar grapes. In this study, the authors assessed growth at 5% (*v*/*v*) ethanol, where the yeast exhibited growth exceeding a 100% yield, while in the study at 8% (*v*/*v*) ethanol, they achieved a growth yield of 80%. When studying stress resistance at a concentration of 13% *v*/*v* ethanol, the cellular growth performance was less than 20%.

Additionally, this yeast possesses other characteristics that contribute to its suitability for the beverage industry. It has been reported that *S. pombe* can assimilate glucose, maltose, sucrose, and raffinose, as well as D-gluconate as a carbon source [43]. It exhibits a remarkable ability to carry out malo-alcoholic fermentation and exceptionally deacidify malic acid [44], with a unique deacidification ability, as most *S. pombe* strains achieve the complete deacidification of malic acid, and have been reported to resist high levels of ethanol, even up to 16% [45]. In addition, it is resistant to preservatives such as sorbic or benzoic acid, with documented tolerance up to 600 mg/L. These characteristics make the yeast a suitable starter for the production of alcoholic beverages, both wines and beers.

Another study conducted by Yao et al. [46] investigated the resistance of *Z. rouxii* to ethanol concentrations of 0%, 5%, 7%, 8%, 9%, and 10% *v*/*v*. These authors reported that the yeast only showed a low cell yield at concentrations of 8%, 9%, and 10% *v*/*v* of ethanol. This finding aligns with our study, as *Z. rouxii* exhibited a meager cell growth rate at the concentration of 8% *v*/*v* ethanol. In the same study, they concluded that, when *Z. rouxii* was cultured alone, survival rates ranged from 6% to 10%, while co-culturing with *T. halophilus* improved the ethanol tolerance of *Z. rouxii*. The tolerance improved with an increase in the co-culturing time.

A similar study was conducted by Fan et al. [47], who evaluated the ethanol tolerance of *C. lusitaniae*. In this study, the yeast maintained an excellent cell growth rate only up to an ethanol concentration of 6% (*v*/*v*). This result is similar to the findings in this work, as *C. lusitaniae* exhibited good cell growth at 5% (*v*/*v*) ethanol but did not perform well fermentatively at 8% (*v*/*v*) ethanol. The authors also reported studies on tolerance to high temperatures of 50 °C, where the yeast could grow over a wide pH range (pH 1–11) and exhibited tolerance to osmotic pressure, growing in media containing 80% glucose. All these results are similar to those obtained in this work when conducting tests for resistance to pH 3.5, high temperatures of 37 °C, and ethanol concentrations of 5% (*v*/*v*) and 8% (*v*/*v*) for the yeast *C. lusitaniae*.

In the study conducted by Boro and Narzary [48], they reported the isolation of two strains of *R. ruineniae*, which they classified as non-amylolytic, non-proteolytic, non-cellulolytic, and non-ethanol producers, with a low tolerance of only 2% (*v*/*v*) ethanol. Compared with this study, *R. ruineniae* exhibited a high growth rate in ethanol stress analyses at concentrations of 5% (*v*/*v*) and 8% (*v*/*v*) ethanol, suggesting that the metabolism of this yeast is strain-dependent.

#### 3.2.3. Hop Tolerance

**Figure 3:**
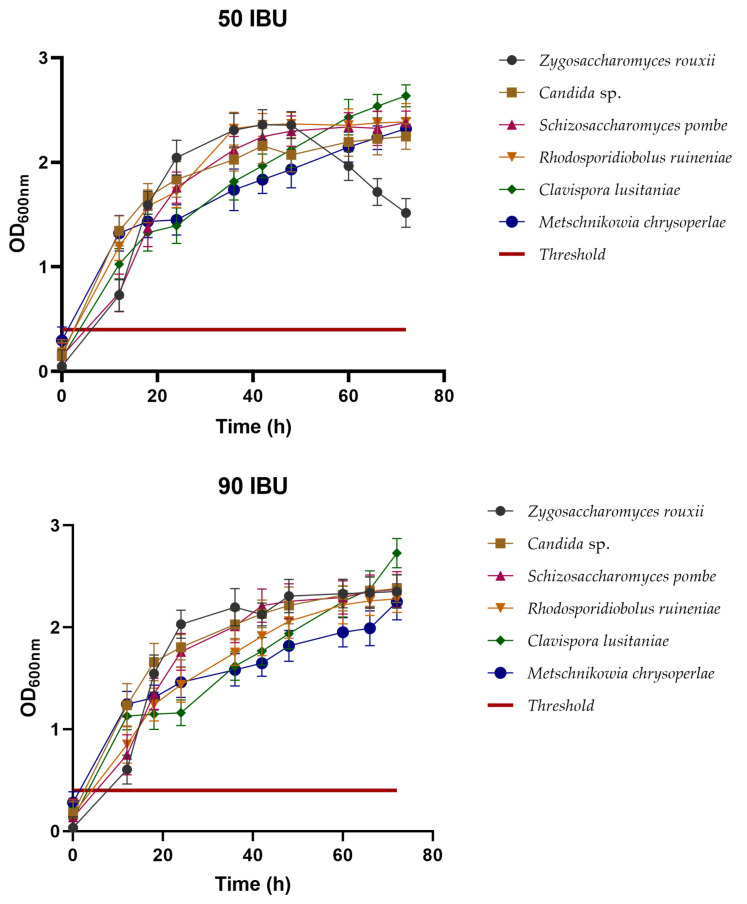
Tolerance to hops at 50 IBU and 90 IBU, represented by the optical density measured at 600 nm in cultures carried out for 72 h. Growth threshold established at 0.4. Values above this point were considered to denote yeast growth, while values below the point were considered to represent the inability of the yeast to grow in the media.

Yeast in the food industry is used in the production of either bread or alcoholic beverages; among these is beer, which is a widely consumed product. In brewing, the addition of hops is an essential step that allows for not only the formation of desirable flavor and bitterness but also allows for the control of other harmful microorganisms. In this sense, the yeasts used for beer making need to have certain tolerance to the compound. Therefore, tolerance to different hop concentrations in a synthetic medium was evaluated. Hop tolerance was assessed using a malt extract medium supplemented with 30% iso-α extract solution (Barth-Haas Group, Nürnberg, Germany). Fermentations were conducted at concentrations of 50 International Bitterness Units (IBU) and 90 IBU. In this study, all yeasts exhibited optimal growth during fermentation, easily surpassing the growth threshold set at 0.4. The behavior was consistent for each treatment (Figure 3). In the 50 IBU fermentation, *C. lusitaniae* showed the highest cellular growth with OD_600_ 2.43.

In comparison, in the 90 IBU fermentation, *Candida* sp. and *S. pombe* exhibited the highest cellular growth, with OD_600_ 2.31 and 2.29, respectively. This suggests that these yeasts would be good candidates for fermenting highly hopped beers, such as IPAs and wheat. Other authors, such as Michel et al. [49], have also reported non-*Saccharomyces* yeasts with good hop tolerance and favorable fermentative characteristics in beer production.

#### 3.2.4. Cross-Tolerance between Ethanol and Hops

**Figure 4:**
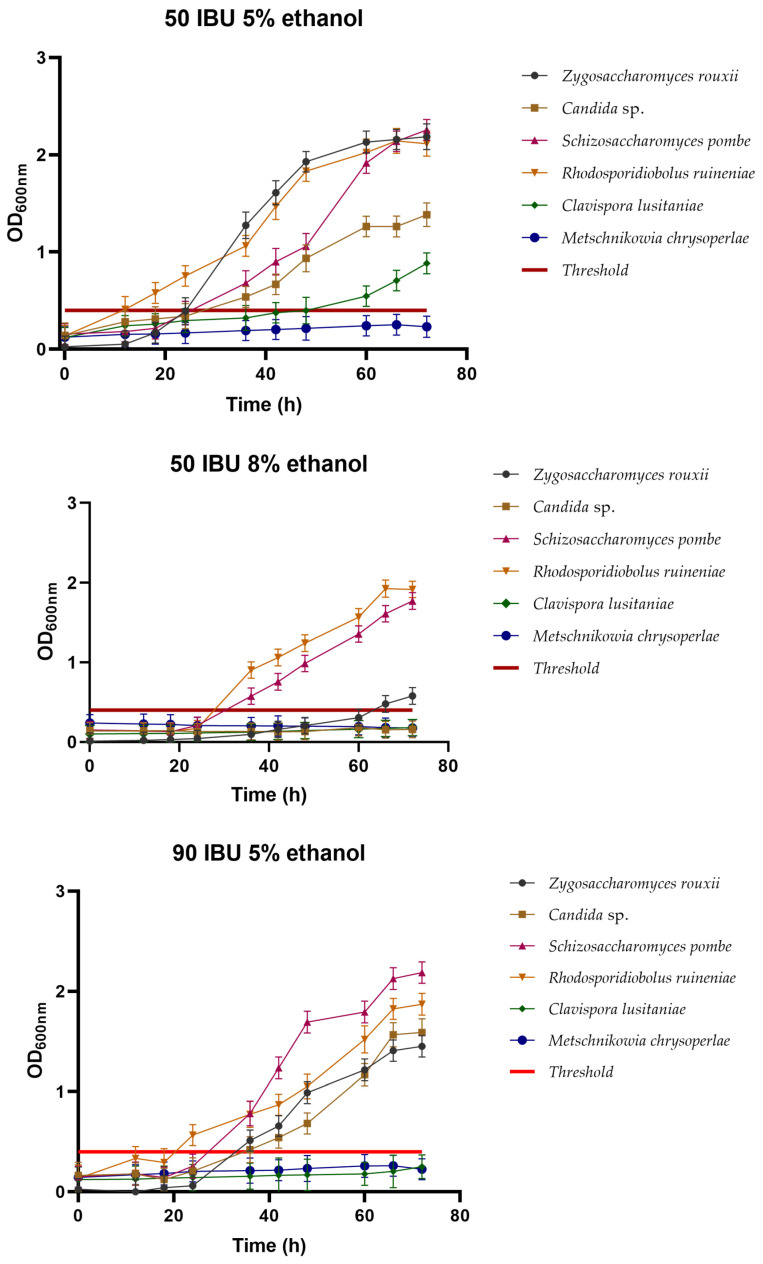

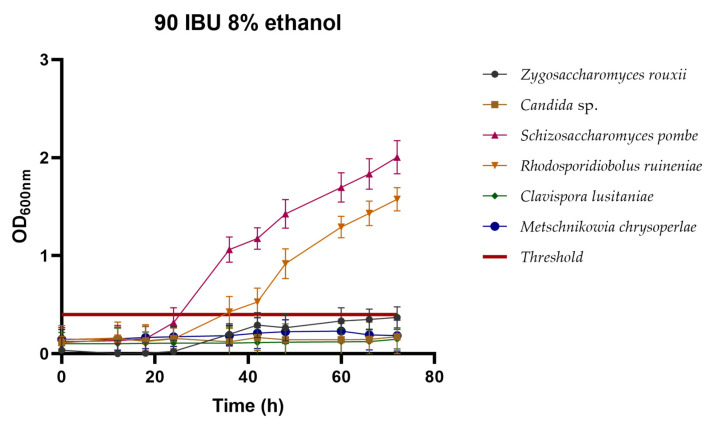
Cross-tolerance to hops and ethanol resistance, represented as the cellular growth determined to 600 nm in cultures carried out for 72 h. Growth threshold established at 0.4. Values above this point were considered to denote yeast growth, while values below the point were considered to represent the inability of the yeast to grow in the media.

To better assess the potential of the isolated yeasts as starter cultures for beer making, a cross-tolerance test was conducted, in which the yeast was subjected to fermentation with both hop and ethanol at different concentrations. Figure 4 shows the results obtained.

In the treatment with 50 IBU + 5% (*v*/*v*) ethanol, the yeasts that exhibited good cellular growth after 72 h were *R. ruineniae* with OD_600_ 2.02, *Z. rouxii* with OD_600_ 1.98, *S. pombe* with OD_600_ 1.91, *Candida* sp. with OD_600_ 1.26, and *C. lusitaniae* with OD_600_ 0.54. *M. chrysoperlae* was the only yeast with a poor fermentative performance, falling below the established growth threshold.

In the case of the treatment with 50 IBU + 8% (*v*/*v*) ethanol, the behavior was different. *S. pombe* and *R. ruineniae* exhibited good fermentative performance with OD_600_ values of 1.35 and 1.56, respectively. Meanwhile, *Z. rouxii* showed poor performance with an OD_600_ of 0.47. Yeasts demonstrating suboptimal fermentative performances included *Candida* sp., *C. lusitaniae*, and *M. chrysoperlae*, falling below the critical growth threshold.

The treatment with 90 IBU + 5% (*v*/*v*) ethanol revealed that the yeasts showing poor fermentative performances were *C. lusitaniae* and *M. chrysoperlae*, with OD_600_ values of 0.17 and 0.25, respectively, falling below the established growth threshold. In contrast, the remaining yeasts *Z. rouxii*, *Candida* sp., *S. pombe*, and *R. ruineniae* demonstrated good fermentative performances, with OD_600_ values of 1.21, 1.16, 1.79, and 1.52, respectively.

In the treatment with 90 IBU + 8% (*v*/*v*) ethanol, the results are different, indicating that the factor affecting the growth of some of these yeasts is ethanol, depending on the concentration used. In this case, the only yeasts that demonstrated good fermentative behavior were *S. pombe* and *R. ruineniae*, with OD_600_ values of 1.69 and 1.29, respectively. Meanwhile, *Z. rouxii*, *Candida* sp., *C. lusitaniae*, and *M. chrysoperlae* exhibited poor fermentative performances, falling below the established growth threshold, with OD_600_ values of 0.36, 0.14, 0.12, and 0.23, respectively.

These results suggest that ethanol would be a determining factor when selecting any of these yeasts. For instance, the behavior of yeasts fermented at 5% (*v*/*v*) ethanol generally showed that *Z. rouxii*, *Candida* sp., *S. pombe*, and *R. ruineniae* demonstrated good fermentative performances, with only *C. lusitaniae* and *M. chrysoperlae* exhibiting poor fermentative behavior under these conditions. However, in the case of the treatment with 8% (*v*/*v*) ethanol, the results were different, and yeasts *S. pombe* and *R. ruineniae* showed high fermentative performances, *Z. rouxii* exhibited lower performance, while *C. lusitaniae* and *M. chrysoperlae* showed poor fermentative performances.

In similar studies conducted by Michel et al. [49], in which they analyzed the ethanol–hops cross-tolerance of non-*Saccharomyces* yeasts, *T. delbrueckii*, isolated from different sources (cheese brine, Pils beer, and sorghum spirits), exhibited good fermentative performance at 50 IBU + 5% (*v*/*v*) ethanol. Similar results were obtained for strains of *T. delbrueckii* isolated from other sources (wheat beer and Pils beer) in the treatment with 90 IBU + 5% (*v*/*v*) ethanol.

In the study conducted by Matraxia et al. [35], a total of 404 yeasts were isolated from fermented honey by products, identified as *Saccharomyces cerevisiae*, *Wickerhamomyces anomalus*, *Zygosaccharomyces bailii*, *Zygosaccharomyces rouxii*, and *Hanseniaspora uvarum*. This work focused on analyzing five strains of *H. uvarum* to assess their beer production capability. Regarding cross-tolerance to ethanol and hops, it was observed that the YGA36 and YGA38 strains were able to grow in the presence of up to 5% ethanol and up to 90 IBU, exhibiting faster growth than the control strain *S. cerevisiae* US-05. The researchers concluded that all strains showed low fermentative power, highlighting especially the ability of the YGA34 strain of *H. uvarum* to overgrow under these stress conditions, making it the selected strain for beer production.

Different studies have assessed the potential of *Z. rouxii*, *S. pombe*, and *M. chrysoperlae* for producing alcoholic beverages. According to the study conducted by Petruzzi et al. [50], *Zygosaccharomyces rouxii* was reported to lack the ability to ferment maltose, making it an ideal candidate for the industrial production of beers with low ethanol levels or non-alcoholic beers. This organism is employed to inhibit alcohol production through biological processes, allowing the creation of innovative, unique beers with intense aromatic profiles. In another study by Callejo et al. [51], it was concluded that *Schizosaccharomyces pombe* was capable of increasing the quantity of volatile compounds such as (1-propanol, isobutanol, 2-methyl-1-butanol, 3-methyl-1-butanol), enhancing the ethanol content, acetaldehyde, and, simultaneously, improving the consistency and persistence of foam, compared to yeasts *Torulaspora delbrueckii*, *Saccharomycodes ludwigii*, and *Lachancea thermotolerans*.

In the study conducted by Liu et al. [52], the behavior of different strains of non-*Saccharomyces* yeasts combined with *Saccharomyces cerevisiae* was analyzed to improve the analytical composition of wines. The authors concluded that three varieties of *Metschnikowia*, one *M. chrysoperlae* and two *M. fructicola*, exhibited a quite similar production of volatile compounds, showing higher levels of isoamyl acetate and (Z)-3-hexenyl acetate than the wine inoculated solely with *S. cerevisiae*. These three wines stood out for their floral and fruity characteristics, with the wine containing *M. chrysoperlae* mainly associated with fruity, tropical, and elderflower notes. *Metschnikowia* strains proved promising for producing Solaris wines with more pleasant flavor profiles.

In the research carried out by Li et al. [53], the performance of sequential fermentation with *Zygosaccharomyces rouxii* and *Saccharomyces cerevisiae* was examined to enhance low-alcohol kiwi wine’s antioxidant activity and aroma. The results of this study indicated that sequential fermentations generated significant increases in total flavonoids, phenols, and antioxidant activity while remarkably improving the aromatic profile. Furthermore, a higher percentage of *Z. rouxii* inoculation contributed significantly to the complexity of volatile compounds, enhancing the sweet aroma of the wines. On the other hand, an equal proportion of inoculation between *Z. rouxii* and *S. cerevisiae* resulted in a significant increase in volatile contents, intensifying the tropical flavor. These findings provide valuable insights into applying non-*Saccharomyces* yeasts in producing innovative beverages. Considering these findings, along with ethanol tolerance, sugar utilization ability, hop resistance, and volatile character, there is a possibility that these yeasts are alternative choices for wine and beer production.

On the other hand, the other three yeasts isolated have limitations for their use in food products. The genus *Candida* sp. Is considered one of the most common endogenous fungi for humans, with *Candida albicans* being the most abundant. Studies have identified the intestinal population of *C. albicans* as one of the main sources of infection [54]. *C. lusitaniae*, also known as *Candida lusitaniae*, is an opportunistic pathogen that infrequently causes invasive candidiasis [55]. *R. ruineniae*, a fungus belonging to the *Basidiomycetes* genus [56], is an opportunistic microbe and an endophyte of various plant species [48]. Therefore, these yeasts were excluded from the probiotic characterization.

### 3.3. Probiotic Potential

In order to assess their probiotic potential, the isolated yeasts were characterized in terms of survival at low pH, growth at body temperature 37 °C, antimicrobial activity, and finally selected isolates were tested to determine its survival through the gastrointestinal passage under simulated conditions.

#### 3.3.1. Survival at Low pH and Growth at 37 °C

**Figure 5:**
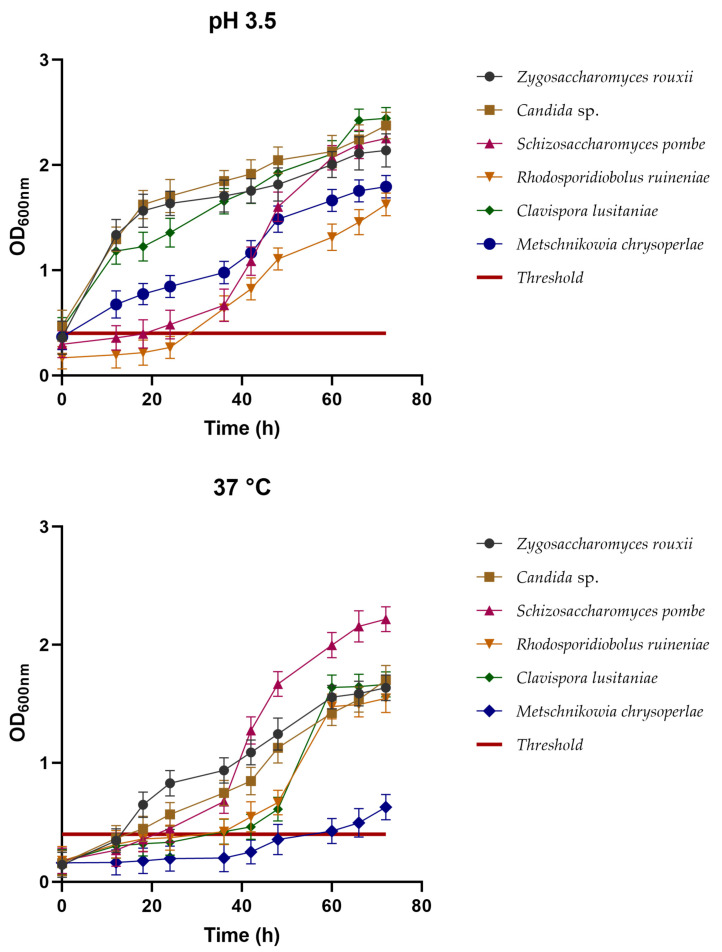
pH tolerance at 3.5 and 37 °C (separately), and cellular growth at 72 h with OD_600_ values for the six isolated yeast strains. Growth threshold established at 0.4. Values above this point were considered to denote yeast growth, while values below the point were considered to represent the inability of the yeast to grow in the media.

Survival at low pH and growth at 37 °C are determining factors to ascertain whether these microorganisms can survive in gastrointestinal conditions, and later grow in the intestine. The six yeast isolates demonstrated good growth when subjected to a pH of 3.5. In Figure 5, it can be observed that these yeasts could tolerate the low pH, maintaining viability after 72 h, with viable counts above 1 × 10^7^ CFU/mL.

Resistance to low pH environments has been previously reported for the studied yeasts isolates. García et al. [42], reported the tolerance of *S. pombe* to pH 3, under aerobic and anaerobic conditions, in which the yeast exceeded a survival of about 80%, similarly to what was observed in our study. Jansen et al. [57] studied the behavior and growth of *Z. rouxii* under different pH values (ranging from three to seven), reporting that the yeast can maintain optimal growth and metabolite production even at the lowest pH values, which are in agreement to what was observed for the isolate studied here. In the case of *R. ruineniae*, Boro and Narzary [48] reported that the yeast is able to grow at a pH range of two to eight. Additionally, these authors reported the yeast’s tolerance to high temperatures, concluding that the maximum survival temperature coincides with the optimal temperature for cell growth at 30 °C. This result differs from the findings in this study, as *R. ruineniae* demonstrated good survival at 37 °C, maintaining a cell count of 1 × 10^7^ CFU/mL. Finally, the pH tolerance of *C. lusitaniae* has been reported before, as stated by the results reported by Ranjan and Sahay [58], in which the yeast was able to growth within a pH range of three to nine.

An important characteristic of probiotic microorganisms is their ability to grow at body temperatures of 37 °C, since this allows their establishment in the intestinal epithelia. All the isolated yeasts were able to surpass the critical limit and showed OD_600_ values above 0.4. However, *S. pombe* showed the best performance, reaching an OD_600_ above 2.0 with a lag phase up to 40 min, and thereafter showed a pronounced exponential phase. Similar behavior was observed for the yeasts *C. lusitaniae* and *R. ruineniae*, which showed a longer lag phase (up to 45 h), and thereafter an exponential one to reach an OD_600_ of about 1.5. Similarly, *Z. rouxii* and the *Candida* isolate showed comparable behavior. The yeasts showed a shorter lag phase and initiated a significant increase in density after the 20 h of experimentation, a trend that was maintained to finally reach a OD_600_ of about 1.5 (Figure 5). *M. chrysoperlae* showed a poor performance, since it was only able to slightly increase its density after 60 h of experimentation.

#### 3.3.2. Auto-Aggregation Capacity

A desirable attribute that a potential probiotic microorganism should possess is the ability to form cell aggregates. This is because such aggregates can enhance the microbe’s adherence to the intestine, thus providing advantages in colonizing the gastrointestinal tract. Auto-aggregation, also known as auto-agglutination or flocculation, is a process that is typically facilitated by self-recognizable surface structures such as proteins and exopolysaccharides, collectively referred to as auto-agglutinins [59]. Although this phenomenon is widespread, its purpose is only sometimes well-understood. Some evidence suggests that the aggregation of bacteria or yeast cells may protect them from environmental stress or host responses. For example, changes in pH levels in the growth medium due to ion exchange or the production of organic acids, ethanol, or antimicrobial compounds, such as killer toxins, can affect the effectiveness with which yeasts can counteract other microorganisms, and cell aggregates can counteract these effects [60].

Auto-aggregation is determined by the formation and sedimentation of yeast or bacterial groups at the bottom of culture tubes. In our study, after 24 h of culture at 37 °C under anaerobic conditions, the percentage of auto-aggregation ranged from 89.8 ± 0.1% for *Z. rouxii* to 100 ± 0.0% for *S. pombe*. Auto-aggregation rates showed considerable variation at 4 h, where values ranged from 3.2 ± 0.0% for *M. chrysoperlae* to 25.8 ± 0.10% for *Candida sp*., indicating that the auto-aggregation capacity is highly influenced by the specific strains under study and the residence time (Table 7). These results are similar to those reported by Gil-Rodríguez et al. [25], who studied the auto-aggregation capacities of the yeasts *S. cerevisiae* IFI-87, *S. pombe* IFI-936, *S. cerevisiae* IFI-244 and *T. delbrueckii* IFI-746.

Auto-aggregation is a phenomenon that normally occurs in the late exponential or stationary phase when sugar is depleted, but it depends on the fermentation mechanism carried out by each yeast [61]. One of the main factors influencing this process is the physicochemical properties of the cell surface, the replicative age of the culture, the presence of Ca^2+^, and certain FLO genes that encode proteins similar to lectins known as adhesins, zymolectins, or flocculants [62]. Cell surface molecules mediate auto-aggregation, which is influenced by the distinct cell wall composition of each strain and the presence of macromolecules extending from the wall, resulting in different behavior for each yeast strain. Compared to bacteria, yeast cells are larger and heavier, causing them to precipitate faster and in greater proportion. All strains selected in this study have an auto-aggregation capacity exceeding 80%, suggesting they possess the described properties. Furthermore, this high capacity may contribute to gastrointestinal tract colonization and forming a physical barrier to pathogen adhesion to mucosal surfaces.

#### 3.3.3. Antimicrobial Activity

Table 8 presents the results of the antimicrobial activity of the isolated yeast strains against *Escherichia coli*, *Staphylococcus aureus*, and *Salmonella enteritidis*. Both culture supernatant and precipitate were studied. In general, the cultures’ precipitate showed a better antimicrobial effect than the supernatant, since, in all the experiments, an inhibition halo was observed. The culture supernatant has the metabolites produced by the yeast, which might have antibacterial properties [63], but they are in low concentrations. On the other hand, the cell precipitate contains lysed cells, which might have also antibacterial properties [64].

All yeasts were able to control the bacteria *S. aureus*. The largest inhibition zone was observed with *M. chrysoperlae* (11.0 ± 0.00 mm) in the yeast cell precipitate assay. The smallest zone was observed when using the *Z. rouxii* yeast precipitate (9.33 ± 0.51 mm). Interestingly, it was observed that the use of the supernatant from the cultures of *S. pombe* had no antibacterial effect, as it did not prevent the growth of *S. aureus*, unlike the assays with precipitates where inhibition zones were observed. Similar results were seen when assays were conducted with *S. enteritidis*; except for *Z. rouxii*, all yeast precipitates could control the growth of the pathogenic bacteria, showing inhibition zones greater than 8 mm. On the other hand, bacterial control using the supernatant was only observed for *M. chrysoperlae*. In the case of *E. coli*, the largest inhibition zone was observed with the precipitate of *M. chrysoperlae*, with inhibition halos of 10.33 mm.

The antimicrobial activity of yeasts belonging to the *Metschnikowia* family has been previously reported. In the study conducted by Savini [65], it was concluded that yeasts of the genus *Metschnikowia* can release pulcherrimina, an antibiotic substance that can inhibit various species of bacteria and fungi. Currently, it is used for pathogen control in post-harvest practices. Pulcherrimina, specifically, is a reddish-toned pigment that can chelate iron ions, immobilizing this metal in the environment. This prevents the growth of antagonized organisms [66,67].

#### 3.3.4. Survival in Simulated *In Vitro* Digestion

Another characteristic that yeasts strains with probiotic potential must possess is the ability to withstand the digestive transit. The three selected yeasts were subjected to *in vitro* gastric and intestinal digestion, following the INFOGEST methodology to assess this. First, the yeasts underwent an oral phase for two minutes with 0.65 mL of simulated oral fluid at pH 7. After two minutes, 5.07 mL of basal gastric fluid with a pH of two was added, initiating gastric digestion for 90 min. During this gastric phase, a syringe pump continuously added 46.35 mL of simulated gastric fluid at a flow rate of 0.515 mL/min for 90 min. *In vitro* digestion involves gastric emptying, corresponding to the passage of chyme from the stomach to the intestine through the pylorus, which acts as a sieve, allowing the passage of liquids and particles smaller than 3 mm [27]. To simulate this biological process, at 18 min intervals during gastric digestion, 20 mL of gastric chyme was collected within 2 min. The chyme was adjusted to pH 7 for use in the subsequent intestinal digestion stage. With the adjusted pH, simulated intestinal fluid was added to the chyme, and intestinal digestion was initiated, maintaining continuous agitation at 150 rpm for two hours at 37 °C. After intestinal digestion, samples were taken for cell counting on YGC agar, incubated at 27 °C for 72 h.

At the end of the *in vitro* digestive process, the three yeast strains studied maintained a viable cell counts at or above 1 × 10^6^ CFU/mL (Table 9), which, as reported by Likotrafiti et al. [68], is the detection limit for the resistance of probiotic strains when submitted to simulated *in vitro* digestion conditions, specifically in the presence of bile salts.

The yeasts *S. pombe* and *M. chrysoperlae* maintained the highest viable cell count of 1 × 10^7^ CFU/mL, being the most resistant to transit through the gastrointestinal tract at the end of the simulated digestion. Meanwhile, the yeast *Z. rouxii* maintained a 1 × 10^6^ CFU/mL viable cell count. All studied yeasts maintained an excellent survival rate, suggesting that these three strains may reach the stomach, survive these conditions, and maintain their viability at intestinal conditions, indicating a potential probiotic character.

Similar results have been reported for two strains of *S. pombe* (IFI-936 and IFI-2180) isolated from foods belonging to the collection of the Food Science Research Institute CIAL (CSIC-UAM). These strains demonstrated good tolerance to gastrointestinal tract conditions and a notable ability to thrive in the intestine, showing robust growth at 37 °C and a high level of auto-aggregation. They also exhibited marked antioxidant activity [69]. The yeast *Z. rouxii* is also described as a microorganism with probiotic characteristics. A commercial strain is currently being evaluated for use in the food and cosmetic industries, due to its probiotic, antioxidant, and antimicrobial capabilities [70]. To our knowledge, this is the first-time probiotic characteristics have been reported for the yeast *M. Chrysoperlae*.

## 4. Conclusions

Our study demonstrated that it is possible to isolate yeasts from Ulmo, Quillay, and Mountain honey, originating from Chile, that exhibit potential probiotic characteristics. Specifically, the yeasts *Zygosaccharomyces rouxii*, *Schizosaccharomyces pombe*, and *Metschnikowia chrysoperlae* were able to tolerate factors such as low pH, bile salts, and temperatures of 37 °C in a simulated *in vitro* digestion system, maintaining cell concentrations above 10^6^ CFU/mL. Additionally, these yeasts could auto-aggregate and showed capabilities for controlling enteric pathogenic bacteria. From the perspective of applications in food production, the yeasts were able to ferment glucose and maltose and produce ethanol as the major metabolite. Good resistance to ethanol and hops was observed in these yeasts, indicating their potential as starter cultures for beer production. This initial screening demonstrates the probiotic and technological potential of these yeasts; however, further studies are needed to determine their technological viability in producing fermented foods and confirm their probiotic characteristics in a food matrix.

## Figures and Tables

**Table 1:** Treatments performed for the phenotypic characterization of the isolated yeasts.

Treatments	Value
Glucose added	10 g/L
Maltose added	60 g/L
pH	3.5
Temperature	37 °C
Ethanol	5% (*v*/*v*) and 8% (*v*/*v*)
Hop	50 IBU and 90 IBU
Ethanol + Hops	50 IBU and 5% (*v*/*v*)
Ethanol + Hops	50 IBU and 8% (*v*/*v*)
Ethanol + Hops	90 IBU and 5% (*v*/*v*)
Ethanol + Hops	90 IBU and 8% (*v*/*v*)

**Table 2:** Components and composition of stock solutions for the preparation of simulated oral, gastric, and intestinal fluids.

Components	Oral Stock Solution (SSO)		Gastric Stock Solution (SSG)		Intestinal Stock Solution (SSI)	
	Amount in Solution	Percentage (%)	Amount in Solution	Percentage (%)	Amount in Solution	Percentage (%)
KCl	3.75	3.75	15.525	1.725	17	1.7
KH_2_ PO_4_	0.925	0.925	2.025	0.225	2	0.2
NaHCO_3_	1.7	1.7	28.125	3.125	106.25	10.625
NaCl	-	-	26.55	2.95	24	2.4
MgCl_2_ (H_2_O)_6_	0.125	0.125	0.9	0.1	2.75	0.275
(NH_4_) 2CO_3_	0.01	0.01	1.125	0.125	-	-
CaCl_2_	0.007	0.007	0.011	0.001	0.11	0.011
H_2_O	93.483	93.483	825.738	91.748	847.89	84.789
Total	100	100	900	100	1000	100

**Table 3:** Components and composition of simulated oral fluid (SOF), gastric fluid (SGF), and intestinal fluid (SIF).

Components	Simulated Oral Fluid (SOF)		Simulated Gastric Fluid (SGF)		Simulated Intestinal Fluid (SIF)	
	Quantity in Solution	Percent (%)	Quantity in Solution	Percent (%)	Quantity in Solution	Percent (%)
Stock solution	8	80	101.76	84.8	100	40
CaCl_2_	0.05	15	0.06	0.05	0.5	0.2
H_2_O	0.45	0.5	5376	4.48	39.5	15.8
α-amylase	1.5	4.5	-	-	-	-
Pepsin	-		8.004	6.67	-	-
HCL (1N)	-		4.8	4	-	-
Pancreatin	-		-	-	62.5	25
N_a_OH (1N)	-		-		10	4
Bile	-		-	-	37.5	15
Total	10	100	120	100	250	100

**Table 4:** Identified yeasts, accession number, and source from which they were isolated.

Yeasts	GeneBank Accession Number	Isolation Source
*Zygosaccharomyces rouxii*	OR392815	Ulmo Honey
*Candida* sp.	OR392816	Ulmo Honey
*Schizosaccharomyces pombe*	OR392817	Ulmo Honey
*Rhodosporidiobolus ruineniae*	OR392818	Mountain Honey
*Clavispora lusitaniae*	OR392819	Mountain Honey
*Metschnikowia chrysoperlae*	OR392820	Quillay Honey

**Table 5:** Analysis of substrates and metabolites in synthetic medium supplemented with glucose 10 g/L.

Yeasts	Glucose (g/L)	Ethanol (% *v*/*v*)	Lactic Acid (g/L)	Acetic Acid (g/L)	Glycerol (g/L)
*Z. rouxii*	0.45 ± 0.002 ^b^	1.56 ± 0.02 ^b^	0.07 ± 0.009 ^b^	0.32 ± 0.02 ^de^	1.68 ± 0.03 ^a^
*Candida* sp.	ND	0.77 ± 0.01 ^d^	0.05 ± 0.006 ^bc^	0.34 ± 0.07 ^d^	0.62 ± 0.005 ^c^
*S. pombe*	ND	2.26 ± 0.04 ^a^	0.23 ± 0.01 ^a^	0.59 ± 0.20 ^b^	1.56 ± 0.06 ^b^
*R. ruineniae*	6.52 ± 0.20 ^a^	0.07 ± 0.008 ^e^	0.04 ± 0.004 ^cd^	0.04 ± 0.20 ^a^	0.35 ± 0.006 ^d^
*C. lusitaniae*	ND	1.12 ± 0.10 ^c^	0.03 ± 0.002 ^d^	0.53 ± 0.01 ^c^	0.21 ± 0.002 ^e^
*M. chrysoperlae*	ND	0.66 ± 0.04 ^d^	0.04 ± 0.006 ^cd^	0.24 ± 0.02 ^e^	0.41 ± 0.008 ^d^

Values represent the average ± standard deviation of triplicate samples. Different lowercase letters in each column represent significant differences (*p* > 0.05) between metabolites produced and/or used by each yeast. ND: not detected.

**Table 6:** Analysis of metabolites and substrates in synthetic medium supplemented with maltose 60 g/L.

Yeasts	Glucose (g/L)	Fructose (g/L)	Sucrose (g/L)	Ethanol (% *v*/*v*)	Lactic Acid (g/L)	Acetic Acid (g/L)	Glycerol (g/L)
*Z. rouxii*	0.54 ± 0.003 ^a^	0.21 ± 0.01 ^a^	4.36 ± 0.06 ^e^	1.17 ± 0.02 ^b^	0.13 ± 0.01 ^b^	0.48 ± 0.05 ^c^	0.66 ± 0.02 ^b^
*Candida* sp.	ND	ND	24.51 ± 0.01 ^b^	0.08 ± 0.007 ^e^	0.05 ± 0.006 ^c^	0.45 ± 0.05 ^c^	ND
*S. pombe*	ND	ND	0.35 ± 0.04 ^f^	1.62 ± 0.06 ^a^	0.27 ± 0.01 ^a^	0.74 ± 0.30 ^ab^	1.17 ± 0.06 ^a^
*R. ruineniae*	ND	ND	15.73 ± 0.03 ^d^	0.62 ± 0.02 ^c^	0.23 ± 0.02 ^a^	0.28 ± 0.04 ^c^	ND
*C. lusitaniae*	ND	ND	17.97 ± 0.30 ^c^	0.6 ± 0.04 ^c^	0.03 ± 0.001 ^c^	0.17 ± 0.03 ^b^	ND
*M. chrysoperlae*	ND	ND	25.91 ± 0.01 ^a^	0.27 ± 0.01 ^d^	0.04 ± 0.005 ^c^	0.59 ± 0.05 ^a^	ND

Values represent the average ± standard deviation of triplicate samples. Different lowercase letters in each column represent significant differences (*p* > 0.05) between metabolites produced and/or used by each yeast. ND: not detected.

**Table 7:** Auto-aggregation capacity for the six yeasts at 24 h.

Yeasts	% Auto-Aggregation
*Z. rouxii*	89.8 ± 0.05%
*S. pombe*	100 ± 0.0%
*M. chrysoperlae*	94 ± 0.01%

Values represent the average ± standard deviation of triplicate samples.

**Table 8:** Antimicrobial activity of the yeast isolates.

Yeasts	*Escherichia coli*		*Salmonella enteritidis*		*Staphylococcus aureus*	
	P (mm)	S (mm)	P (mm)	S (mm)	P (mm)	S (mm)
*Z. rouxii*	9 ± 0.10	9 ± 0.0	-	-	9.33 ± 0.51	10.66 ± 0.52
*S. pombe*	9 ± 0.0	9.33 ± 0.50	9.66 ± 0.50	-	9.66 ± 1.10	-
*M. chrysoperlae*	10.33 ± 0.50	10 ± 0.0	9.33 ± 1.10	9 ± 1.05	11 ± 0.0	10 ± 0.10

The values represent the mean ± standard deviation of triplicate samples. The are no significant differences (*p* > 0.05) in the antimicrobial activity for each yeast. P: precipitate. S: supernatant.

**Table 9:** Cell count after each phase of *in vitro* digestion.

Yeasts	Cell Concentration (CFU/mL)		
	Oral Phase	Gastric Phase	Intestinal Phase
*Z. rouxii*	1 × 10^9^ ± 0.65	1 × 10^9^ ± 0.37	1 × 10^6^ ± 0.63
*S. pombe*	1 × 10^9^ ± 0.32	1 × 10^9^ ± 0.52	1 × 10^7^ ± 0.12
*M. chrysoperlae*	1 × 10^9^ ± 0.56	1 × 10^8^ ± 0.71	1 × 10^7^ ± 0.25

The values represent the mean ± standard deviation of triplicate samples.

## Data Availability

The original contributions presented in the study are included in the article, further inquiries can be directed to the corresponding author.
